# Diabetes mellitus in Zambia and the Western Cape province of South Africa: Prevalence, risk factors, diagnosis and management

**DOI:** 10.1016/j.diabres.2016.05.001

**Published:** 2016-08

**Authors:** Sarah Lou Bailey, Helen Ayles, Nulda Beyers, Peter Godfrey-Faussett, Monde Muyoyeta, Elizabeth du Toit, John S. Yudkin, Sian Floyd

**Affiliations:** a LSHTM TB Centre and Department of Clinical Research, London School of Hygiene and Tropical Medicine, Keppel Street, London, UK; b Zambart, Ridgeway Campus, Lusaka, Zambia; c Desmond Tutu TB Centre, Department of Paediatrics and Child Health, Stellenbosch University, South Africa; d University College London, Gower Street, London, UK; e LSHTM TB Centre and Department of Infectious Disease Epidemiology, London School of Hygiene and Tropical Medicine, Keppel Street, London, UK

**Keywords:** Southern Africa, Cross-sectional study, Epidemiology

## Abstract

**Aims:**

To determine the prevalence of and risk factors for diabetes mellitus and examine its diagnosis and management in the study communities.

**Methods:**

This is a population-based cross-sectional study among adults in 24 communities from Zambia and the Western Cape (WC) province of South Africa. Diabetes is defined as a random blood glucose concentration (RBG) ⩾ 11.1 mmol/L, or RBG < 11.1 mmol/L but with a self-reported prior diabetes diagnosis. For individuals with a prior diagnosis of diabetes, RBG < 7.8 mmol/L was considered to be an acceptable level of glycaemia.

**Results:**

Among 45,767 Zambian and 12,496 WC participants the age-standardised prevalence of diabetes was 3.5% and 7.2% respectively. The highest risk groups identified were those of older age and those with obesity. Of those identified to have diabetes, 34.5% in Zambia and 12.7% in WC were previously unaware of their diagnosis. Among Zambian participants with diabetes, this proportion was lower among individuals with better education or with higher household socio-economic position. Of all those with previously diagnosed diabetes, 66.0% in Zambia and 59.4% in WC were not on any diabetes treatment, and 34.4% in Zambia and 32.7% in WC had a RBG concentration beyond the recommended level, ⩾7.8 mmol/L.

**Conclusions:**

The diabetes risk factor profile for our study communities is similar to that seen in high-income populations. A high proportion of individuals with diabetes are not on diabetes treatment and of those on treatment a high proportion have high glycaemic concentrations. Such data may assist in healthcare planning to ensure timely diagnosis and management of diabetes.

## 1 Introduction

The number of adults with diabetes mellitus in sub-Saharan Africa (SSA) is predicted to rise from 19.8 million in 2013 to 41.5 million in 2035 [1], [2], [3], [4], [5]. Extensive data exist to guide diabetes public health policies and health systems planning in high income countries [6], [7], [8]. In contrast, systematic review suggests there are few data from SSA even for diabetes prevalence and risk factors [1], [9], [10], [11], [12].

The management of diabetes can be challenging for health systems, as it requires lifelong follow-up and a multidisciplinary approach [3], [13], [14]. Therefore, the aims of this study are

• to estimate the prevalence of, and identify risk factors for, diabetes mellitus in the study communities in Zambia and in the Western Cape province of South Africa; and
• to estimate the prevalence of undiagnosed diabetes, to determine the proportion of those with a prior diagnosis of diabetes who are on treatment for diabetes, and to determine the levels of glycaemia in those with a prior diagnosis of diabetes.

## 2 Methods

This population-based cross-sectional study was undertaken between January and December 2010 in 24 communities: 16 from 5 provinces in Zambia and 8 from the Western Cape province of South Africa. The study was nested into a prevalence survey that was conducted to measure the primary endpoint, prevalent tuberculosis, of a large 2 × 2 factorial cluster randomised trial (the ZAMSTAR study) [15], [16], [17]. The estimated total population in the study areas was 962,655, with an average population per community of 40,110. Within each community, a two-stage cluster sampling design was used to recruit participants. Exclusion criteria were age <18 years, inability to give informed consent due to disability/incapacitation, refusal to submit a respiratory sample – for purposes of the parent study – and any persons living in institutional settings.

Each participant was required to give written informed consent. Individuals and household heads were interviewed in their homes using structured questionnaires. Finger prick capillary blood was taken for HIV testing and random blood glucose (RBG) measurement, with pre- and post-test counselling for HIV tests. Determine ™HIV-1/2 was used for HIV testing, plus UniGold™HIV-1/2 to confirm all positives. RBG concentration was measured using an Optium Xceed point-of-care glucometer. All research staff were trained on the use of this particular glucometer and were required to undergo proficiency testing. Standardised control solution was used for performance checks on test strips and meters. Height and weight were measured using standard operating procedures. All individuals identified to have abnormal blood glucose or to be HIV positive were referred to existing local health facilities for appropriate management.

Data were electronically entered directly onto personal digital assistants by field staff at the time of data collection, using pre-programmed questionnaires and result sheets. All information was downloaded daily into a SQL (structured query language) database and later exported into Stata.

Ethics approval was granted from the London School of Hygiene and Tropical Medicine Ethics Committee, the University of Stellenbosch Ethics Committee and the University of Zambia Ethics Committee.

### 2.1 Definitions

• Diabetes mellitus was defined as a random blood glucose concentration (RBG) ⩾ 11.1 mmol/L, or RBG < 11.1 mmol/L but with a self-reported prior diabetes diagnosis.
• Body mass index (BMI) was defined as weight in kilograms divided by height squared in metres (weight(kg)/height^2^(m)).
• HIV status was defined by a combination of blood sampling and self-report for those with missing biological data.
• Exposures (risk factors) for diabetes were defined as proximal or distal factors. Distal factors include age, sex, household socio-economic position, education, smoking history, HIV status and community. The proximal factor, BMI, may be determined partly by the distal factors and so estimation of its direct effect on diabetes requires controlling for confounding by the more distal factors.
• For assessing the management of those with diabetes, RBG < 7.8 mmol/L was considered to be the recommended level of glycaemia, as specified by the International Diabetes Federation guideline for target postprandial glucose concentration [18].

### 2.2 Statistical analyses

Direct age standardisation for the prevalence of diabetes was calculated by applying the study age-specific diabetes rates (separately for Zambia and the Western Cape) to the 2013 International Network for the Demographic Evaluation of Populations and Their Health (INDEPTH) sub-Saharan African standard population distribution. Univariable and multivariable logistic regression analyses were used to identify risk factors for diabetes, accounting for the cluster sampling design. Principal components analysis was used to create a measure of household socio-economic position separately for each country, using the following variables: main type of dwelling; main type of flooring; main type of household toilet; main source of household drinking water, and presence of household assets including radio, television, refrigerator, bicycle, motorcycle, car, domestic worker and mobile phone.

The variables considered *a priori* as potential risk factors were those known to be risk factors in other populations and settings [19], [20]: age, sex, household socio-economic position, education, smoking, ethnicity and adiposity (measured by BMI). HIV status was also considered given its high prevalence in both localities, and the known effects of some antiretroviral medications on insulin resistance [21]. Rural/urban location was not considered as a binary variable as a finer categorisation of location was required to account for between-community variation in analysis of household and individual-level risk factors.

Household socio-economic position and individual education are expected to be inter-related. For the analyses presented here, education was considered to be the more distal variable and we explored whether the association between education and diabetes prevalence was partly mediated by household wealth.

Body mass index was considered to be a factor that is proximal to household socio-economic position and education on the causal pathway to diabetes, and also to age, sex and HIV status. To estimate the association of these more distal factors with the outcome (to identify high risk groups) it was important first to exclude the measure of obesity from multivariable analyses, and secondly to include it so as to identify how much of any observed associations were mediated by BMI. Therefore, separate final models were created, with and without inclusion of BMI. All data analyses were performed using Stata12.

## 3 Results

**Fig. 1:**
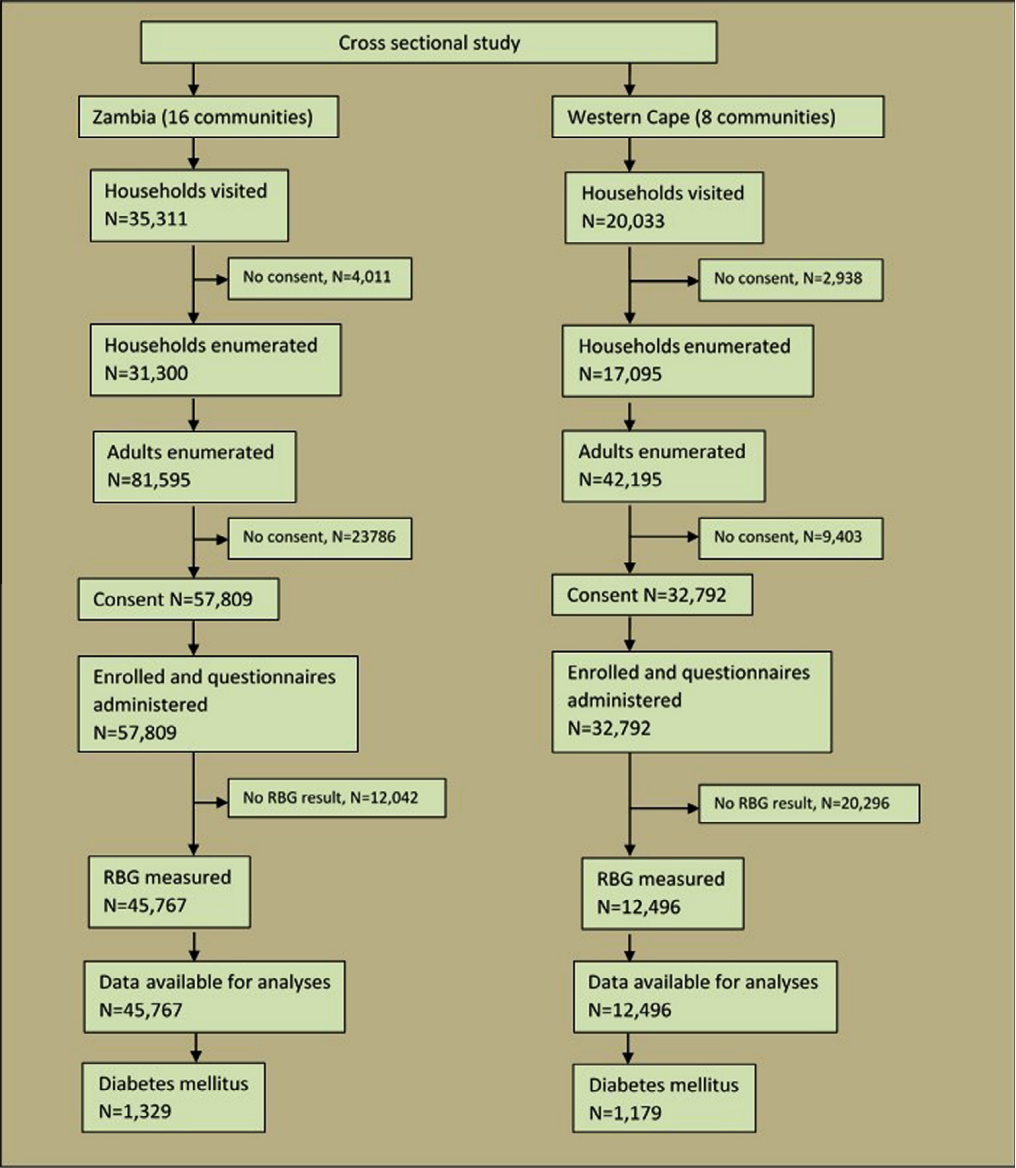
Number and flow of study participants and cases in Zambia and the Western Cape of South Africa.

In Zambia, 57,809 (70.8% of eligible) participants from 31,300 (88.6% of eligible) households were enrolled. In Western Cape, 32,792 (77.7% of eligible) participants from 17,095 (85.3% of eligible) households were enrolled (Fig. 1). Complete RBG results were obtained for 45,767 (79.2% of enrolled) participants in Zambia and 12,496 (38.1% of enrolled) participants in Western Cape. Among all participants with an RBG measurement (those forming the final dataset for analyses) 524 (1.1%) in Zambia and 9 (0.1%) in Western Cape had missing data for age, 3883 (8.5%) and 788 (6.3%) respectively had missing data for HIV status, and 3536 (7.7%) and 365 (2.9%) respectively had missing data for BMI.

The distribution of the baseline characteristics for all participants who contributed to the analyses is shown in Table 1, Table 2. The study participants ranged between ages 18–102 (mean 33.3 years) years in Zambia and 18–103 years (mean 37.0 years) in Western Cape. Two-thirds of the participants were female in both countries. The distribution of the baseline characteristics for participants with missing glycaemia data are given in a supplementary table. The only large difference in distribution of individuals with and without glycaemia data occurred for the community variable.

### 3.1 Prevalence

**Fig. 2:**
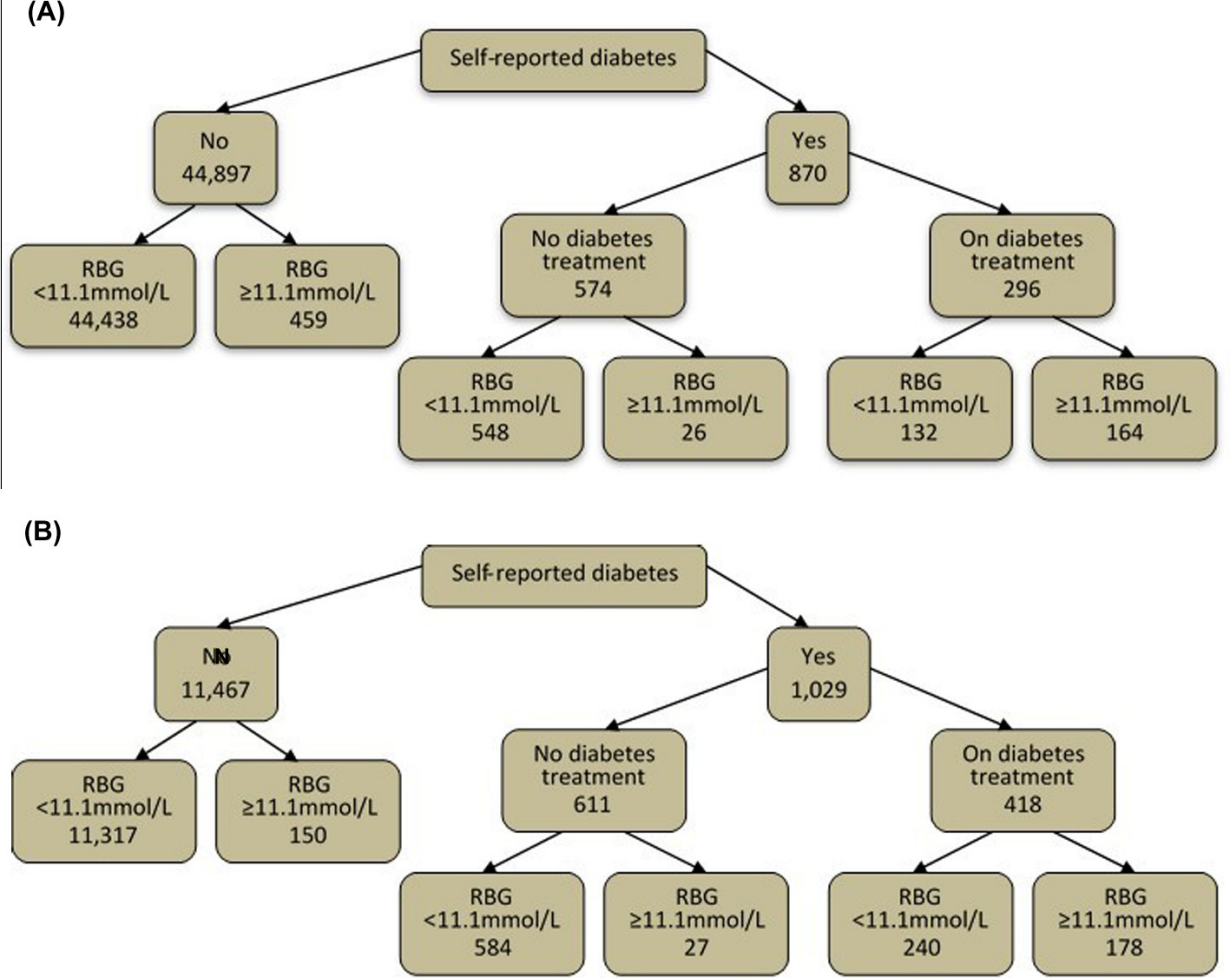
Numbers of (a) Zambian and (b) Western Cape participants with diabetes by self-report and RBG diagnosis, and numbers who report they are currently on treatment for diabetes. Legend: treatment = dietary, oral hypoglycaemic agents or insulin.

Fig. 2 shows the numbers of people with diabetes according to self-report and positive screen. In Zambia 65.5% (870/1329) of people with diabetes were previously diagnosed whereas in Western Cape the figure was 87.3% (1029/1179). The prevalence of diabetes mellitus among study participants in Zambia was 2.9% and in Western Cape 9.4%. Diabetes prevalence stratified by baseline characteristics is shown in Table 1, Table 2. The prevalence was highest in older participants and in those with a higher body mass index.

The INDEPTH sub-Saharan African standard population age-standardised prevalence of diabetes mellitus was 3.5% for the Zambian communities and 7.2% for the Western Cape communities.

### 3.2 Risk factors

After multivariable analyses (Table 1, Table 2), the distal risk factors for diabetes mellitus identified in these study populations were age, sex, household socio-economic position, HIV status, and community for Zambia, and age, sex, smoking, HIV status and community for the Western Cape communities, with age being the strongest predictor in both settings. In Zambia the association between education and diabetes was strongly confounded by age; after adjusting for age the odds of diabetes increased with education level, and the strong confounding was due to the better educated being younger, on average. The odds of diabetes also increased with higher household socio-economic position in Zambia, and this explained some of the association between education and diabetes (ORs for education grade 3–6, 7–10, 11–12, and College/University were 1.22, 1.39, 1.62, and 1.87, *p* < 0.001, with adjustment for age, sex, community, smoking and HIV but not household socio-economic position). Ethnicity was not identified as a risk factor and was not included in the final multivariable model as it varied little within communities in the Western Cape so that community and ethnicity effects could not be distinguished, and varied little across all the Zambian communities.

There was strong evidence that the proximal risk factor, BMI, was associated with diabetes in both Zambia and the Western Cape communities. Comparison of the models with and without controlling for BMI shows that after adjustment for BMI the higher odds of diabetes among women compared with men is no longer seen for Zambian participants (Table 1) and is slightly reduced in Western Cape participants (Table 2). The association seen between smoking history and diabetes in Western Cape participants is also reduced. The associations with age in both countries, household socio-economic position in Zambian communities, and HIV status in Western Cape communities remain after accounting for the measure of adiposity, but are all reduced in magnitude.

When HIV status is further sub-divided by current use or not of antiretroviral therapy (ART), the odds of diabetes in Western Cape communities is lowest in the group who are HIV positive but not on ART: ORs for HIV negative, HIV positive not on ART and HIV positive on ART were 1, 0.69, and 1.03, *p* = 0.006, with adjustment for all proximal and distal factors. No association remains for the Zambian communities.

Using self-report rather than the RBG-based definition used for the rest of this study removes potential biases resulting from the use of RBG as the measurement tool and simultaneously increases the number of participants without missing data in the fully adjusted models to 43,060 in Zambia and 11,508 in the Western Cape. Of note, fitting the same multivariable models but with the outcome of diabetes defined entirely by self-report showed that odds ratios and evidence for associations were similar to the findings summarised in Table 1, Table 2. Likewise, redefining diabetes to include only those with RBG ⩾ 11.1 mmol/L plus those on treatment for diabetes regardless of their RBG concentration confirms the main risk factor profile identified. This definition removes the group who have self-reported diabetes but are not on treatment for diabetes and have a RBG < 11.1 mmol/L. Age and BMI are again identified as the main risk factors for diabetes, and sex is no longer identified as a risk factor in Western Cape.

### 3.3 Diagnosis and management

Fig. 2 shows for Zambia and Western Cape the numbers of participants with diabetes by self-report and RBG diagnosis, along with numbers on treatment for diabetes.

The prevalence of undiagnosed diabetes among the total study population, including among those without diabetes, was 1.0% in the Zambian sites and 1.2% in the Western Cape sites. Stratification by participant characteristics for the prevalence of undiagnosed diabetes among all those with diabetes is shown in Table 3. Strong evidence for unadjusted association with undiagnosed diabetes is seen for household socio-economic position, education and community in the Zambia sites, and age and community in the Western Cape sites.

Of all those who had a prior diabetes diagnosis, 34.0% in Zambia and 40.6% in Western Cape were on diabetes treatment consisting of either dietary management, hypoglycaemic tablets or insulin therapy (Table 4). Among those with a prior diagnosis who were on diabetes treatment, the mean random blood glucose concentration for Zambian participants was 12.8 mmol/L (standard deviation, SD, 6.6 mmol/L). The corresponding mean for Western Cape participants was 11.0 mmol/L (SD 5.8 mmol/L). Among those with a prior diagnosis who were not on any diabetes treatment, the mean RBG concentration for Zambian participants was 6.4 mmol/L (SD 3.0 mmol/L) and for Western Cape participants was 6.3 mmol/L (SD 2.7 mmol/L). Table 5 shows these data as numbers of participants by category of RBG concentration.

## 4 Discussion

The prevalence of diabetes mellitus in these study communities rivals that seen in high-income settings [22], [23]. After adjustment for confounding factors, there is strong evidence that the prevalence of diabetes in participants from both countries increases with age and BMI, and differs by community. Among Zambian participants there is strong evidence that the prevalence of diabetes increases with increasing household socio-economic position, and also with level of education attained when the interrelated effect of household socio-economic position is not adjusted for.

Among Western Cape participants there is strong evidence that the prevalence of diabetes is higher in females than males and among those who are HIV negative compared to those who are HIV positive. Participation was higher in women than men in all study communities, most likely because women were more readily found in their homes, the site of enrolment, than men. The same was true for participants who were excluded from the analyses due to missing glycaemia data (supplementary table) and so is unlikely to have caused any systematic bias.

BMI, as a measure of adiposity, entirely explains the male–female differential in diabetes prevalence in the Zambian sites and most of the association with smoking in the Western Cape sites, but only partially explains associations with age, education and household socio-economic position. These distal factors are therefore associated with diabetes independently of adiposity, in addition to the mediating pathway via adiposity. The remaining association with age is explained by differences in self-reporting of diabetes between men and women: the association is removed entirely when the diabetes definition used removes the group who report that they have diabetes but have RBG < 11.1 mmol/L and are not on treatment for diabetes.

Of those with diabetes, individuals who are more likely to have undiagnosed diabetes are those who are older, from a lower household socio-economic position and with a lower level of education. Of those with self-reported previously diagnosed diabetes, many remain on no treatment for their diabetes, not even dietary management. A high proportion have high random blood glucose concentrations, though more so among those who are on treatment for their diabetes rather than among those who are not on treatment. This could indicate that those with the most poorly controlled diabetes are more likely to be given treatment, even though the treatment they are given is sub-optimal, or it could reflect measurement error for self-reported prior diabetes diagnosis.

The risk factor profile seen in this study is similar to that seen for diabetes elsewhere except for the pattern of association seen with HIV status in the Western Cape [11], [12], [19], [24], [25], [26]. A reduced risk of diabetes for those with HIV but not on antiretroviral therapy is unexpected. It would be more understandable for those with HIV and on anti-retroviral medication to have an enhanced risk of diabetes, due to anti-retroviral effects on insulin resistance. The association is partially explained by adiposity: HIV can cause a reduction of adiposity, particularly when not on ART, and consequently a reduction of diabetes risk. Residual confounding by central obesity is a possible explanation but beyond this the association is most likely explained by chance, other residual confounding, or bias.

Diabetes is well known to be associated with urban/rural location [11], [12], [24], [25]. Although not formally explored, an assessment of this can be made in these data through the community variable. For both study populations no clear urban/rural pattern is seen. This could be explained by the nature of the rural communities that were chosen to take part in the ZAMSTAR cluster-randomised trial. These communities are mostly central areas of rural districts rather than remote villages, and so may not be so different from the urban areas.

The prevalence of diabetes was found to be higher in the Western Cape communities than in the Zambian communities, even for comparable age, sex, relative household socio-economic position and education, and BMI. This could be due to differences in dietary habits, levels of physical activity or genetic factors between the two localities, but as the study was not designed to explore this we are unable to draw definitive conclusions on the cause of the difference in prevalence between the two settings.

Misclassification of outcome data could have resulted in bias. For diabetes, an under-estimate of association is likely as RBG tests have good specificity but sub-optimal sensitivity for diabetes diagnosis [27], [28]. It is reassuring for the determination of risk factors, that almost identical associations were seen when diabetes diagnosis was defined in other ways. However, for both the prevalence of diabetes and the prevalence of undiagnosed diabetes the figures determined in this study likely represent the minimum true proportions. Indeed, the proportion of individuals with diabetes who are undiagnosed has been reported to be much higher in other parts of Africa, up to 75% in Northern Africa [13]. The proportion observed among our Western Cape participants is even comparable to that reported from the United States [29]. It is possible that the comparatively low proportion of the total study population who have undiagnosed diabetes is partly due to a low sensitivity of RBG ⩾ 11.1 mmol/L for diabetes diagnosis.

Alternative approaches to the study methods could have been to measure fasting blood glucose or glycated haemoglobin concentrations, or to perform oral glucose tolerance tests. Any of these methods would have resulted in greater sensitivity for diabetes diagnosis, but in a large-scale field study with data collection occurring in the community each of these approaches would have been logistically challenging and likely less acceptable to potential participants. Consequently the likelihood of measurement error and a low uptake of potential participants would have been high. Therefore it was felt that the use of RBG measurement for this study would optimise participant uptake and minimise measurement error.

Even with this approach, the number of participants with missing data for RBG concentration was substantial. However, given that the primary focus of data collection was tuberculosis prevalence, for the purposes of the parent study, these losses are more likely to be due to their lack of prioritisation during the data collection process rather than due to a lack of acceptability to participants. Further, in Western Cape participants were required to attend a mobile clinic for capillary blood tests whereas in Zambia the tests were performed in participants’ homes, which most likely is the explanation for the higher proportion of missing RBG results seen in Western Cape than Zambia. For these reasons the potential for the missing data to cause bias to the study results, through being associated with glycaemic concentration, is low.

The accuracy of RBG results obtained in our study is a consideration. Although point-of-care capillary blood glucose measurement is more rapid, cost-effective and less invasive than laboratory measurement of plasma glucose concentration, the latter is considered to be the most accurate method. Although performance checks were made on test strips and meters, we have limited quantitation of glucometer characteristics obtained during data collection for this study. Accuracy data from other settings are reassuring: a recently reported study comparing six commonly used point-of-care blood glucose monitoring systems found the Optium Xceed system to have the highest level of accuracy, the lowest occurrence of error messages and to be least influenced by blood haematocrit levels [30]. The Optium Xceed system met current accuracy criteria set by the International Organization for Standardization, having >95% of all readings within ±12.5% from the reference at glucose levels >4 mmol/L and ±0.5 mmol/L at glucose levels <4 mmol/L.[30] Consideration of test accuracy is most relevant to prevalence estimates obtained in this study as inaccurate results could have led to estimates that are either too high or too low. However, there is no reason that glucometer performance would vary systematically by age or gender or other participant characteristic, and so inaccurate performance would only weaken associations between participant characteristics and glucose result. It is reassuring, therefore, that the main risk factors identified in this study are known to be established risk factors for diabetes elsewhere [19], [20].

The use of random blood glucose concentration to determine satisfactory control of glycaemia is a major limitation. When interpreting the proportions in this study of those with a prior diabetes diagnosis who were found to have an inadequate level of glycaemia, it is again important to appreciate that this is based on a one-off measurement of random blood glucose concentration, not on a measure of longer-term glycaemic control such as glycated haemoglobin. In this large-scale population-level study setting the measure used and results obtained are certainly suggestive that glycaemic control is sub-optimal, but interpretation beyond this should be made only with caution.

As participants were sampled at random and in sufficiently large numbers, it is possible to generalise these results to the communities from where the participants came. Generalisation beyond this should be made with caution, as the communities were not selected at random from the wider population.

## 5 Conclusions

The prevalence of diabetes mellitus in the study communities rivals that seen in high-income settings. The risk factor profile is similar to that seen in Caucasian populations, with age ⩾50 years and BMI ⩾ 30 representing the highest risk groups for diabetes. The study findings suggest that many of those with diabetes remain undiagnosed in the community, particularly among those from a lower socio-economic position and with a lower level of education in the Zambian sites. Even if diagnosed, many of those with diabetes remain sub-optimally managed. Further studies to guide effective methods of managing diabetes at the individual and public health levels in low-income sub-Saharan settings are needed. Timely diagnosis and management of this long-term non-communicable disease must be prioritised, with a particular emphasis on redressing the lack of health equity for the poorer and less educated.

## Author contributions

All authors contributed to initial study concept and study design. HA, NB and PGF are the principal investigators of the ZAMSTAR study, into which this study is nested. They oversaw participant recruitment and data collection. SLB and SF performed the data analysis. SLB wrote initial drafts and all authors contributed to final editing of the paper.

## Conflicts of interest

We declare that we have no conflicts of interest.

## Funding

SLB is supported by a Wellcome Trust Clinical PhD Fellowship (100141/Z/12/Z). The ZAMSTAR study was supported by a subcontract from Johns Hopkins University with funds provided by Grant No. 19790.01 from the Bill and Melinda Gates Foundation. The contents of this manuscript are solely the responsibility of the authors and do not necessarily represent the official views of the Bill and Melinda Gates Foundation or the Wellcome Trust.

## Figures and Tables

**Table 1:** Prevalence of diabetes mellitus in the Zambian sites, with corresponding unadjusted and adjusted odds ratios estimated by logistic regression, showing adjustment for distal risk factors (without measure of adiposity) and more proximal factor (body mass index).

Characteristic		Total number (%)	Number (%) with DM⁎	Unadjusted OR (95% CI)	*P*-value#	Adjusted OR excluding BMI (95% CI)+	*P*-value#	Adjusted OR including BMI (95% CI)++	*P*-value#
Overall		45,767 (100)	1329 (2.9)						
Age (years)	18–24	15,983 (35.3)	202 (1.3)	1	<0.001 (TFT *p* < 0.001)	1	<0.001 (TFT *p* < 0.001)	1	<0.001 (TFT *p* < 0.001)
	25–29	7590 (16.8)	107 (1.4)	1.09 (0.86–1.39)		1.12 (0.88-1.43)		1.04 (0.81–1.35)	
	30–34	5583 (12.3)	122 (2.2)	1.70 (1.35–2.13)		1.73 (1.36–2.21)		1.53 (1.19–1.98)	
	35–39	3971 (8.8)	106 (2.7)	2.23 (1.76–2.84)		2.38 (1.84–3.07)		2.08 (1.59–2.73)	
	40–49	5195 (11.5)	232 (4.5)	3.80 (3.13–4.61)		3.89 (3.14–4.81)		3.03 (2.40–3.81)	
	50–59	3540 (7.8)	277 (7.8)	7.19 (5.95–8.69)		7.50 (6.08–9.26)		5.86 (4.67–7.36)	
	60+	3381 (7.5)	256 (7.6)	7.23 (5.96–8.77)		7.93 (6.27–10.03)		6.79 (5.29–8.72)	

Sex	Male	15,153 (33.1)	403 (2.7)	1	0.022	1	0.010	1	0.893
	Female	30,614 (66.9)	926 (3.0)	1.15 (1.02–1.30)		1.22 (1.05–1.43)		0.99 (0.84–1.17)	

Household socio-economic position	Very low	10,571 (23.1)	229 (2.2)	1	<0.001 (TFT *p* < 0.001)	1	<0.001 (TFT *p* < 0.001)	1	<0.001 (TFT *p* < 0.001)
	Low	12,058 (26.4)	331 (2.8)	1.22 (1.01–1.46)		1.31 (1.07–1.60)		1.23 (1.00–1.53)	
	Medium	11,570 (25.3)	366 (3.2)	1.46 (1.20–1.77)		1.60 (1.29–1.98)		1.45 (1.16–1.83)	
	High	11,568 (25.3)	403 (3.5)	1.83 (1.46–2.30)		2.02 (1.57–2.62)		1.79 (1.37–2.34)	

Highest level of education	None/grade 1-2	3507 (7.7)	142 (4.1)	1	<0.001 (TFT *p* < 0.001)	1	0.074 (TFT *p* = 0.004)	1	0.089 (TFT *p* = 0.008)
	Grade 3-6	5753 (12.6)	191 (3.3)	0.81 (0.65–1.02)		1.21 (0.92–1.58)		1.26 (0.94–1.68)	
	Grade 7–10	22,486 (49.1)	642 (2.9)	0.61 (0.50–0.73)		1.30 (1.01–1.66)		1.35 (1.04–1.77)	
	Grade 11–12	9947 (21.7)	201 (2.0)	0.40 (0.32–0.51)		1.42 (1.06–1.92)		1.49 (1.09–2.05)	
	College/University	4074 (8.9)	153 (3.8)	0.72 (0.57–0.93)		1.56 (1.14–2.14)		1.56 (1.12–2.18)	

Smoking history	Never	39,743 (86.8)	1170 (2.9)	1	0.583 (TFT *p* = 0.334)	1	0.169 (TFT *p* = 0.071)	1	0.397 (TFT *p* = 0.194)
	Ex-smoker	2319 (5.1)	70 (3.0)	0.99 (0.77–1.28)		0.96 (0.72–1.27)		0.97 (0.73–1.29)	
	Current smoker	3705 (8.1)	89 (2.4)	0.89 (0.71–1.11)		0.78 (0.59–1.02)		0.83 (0.63–1.09)	

HIV status⁎⁎	Negative	36,643 (82.7)	947 (2.7)	1	0.169	1	0.041	1	0.646
	Positive	7241 (17.3)	202 (2.8)	0.90 (0.77–1.05)		0.84 (0.71–0.99)		0.96 (0.81–1.14)	

Body Mass Index⁎⁎⁎ (weight(kg)/height^2^(m))	Recommended weight (18.5–24.9)	26,865 (63.6)	537 (2.0)	1	<0.001 (TFT *p* < 0.001)##			1	<0.001 (TFT *p* < 0.001)##
	Underweight (<18.5)	4203 (10.0)	98 (2.3)	1.12 (0.90–1.40)				1.09 (0.86–1.38)	
	Overweight (25–29.9)	7429 (17.6)	327 (4.4)	2.26 (1.96–2.61)				1.52 (1.29–1.79)	
	Obese (⩾30)	3734 (8.8)	268 (7.2)	3.82 (3.27–4.46)				2.29 (1.89–2.77)	

Community	ZAM1	3675 (8.0)	282 (7.7)	1	<0.001	1	<0.001	1	<0.001
	ZAM2	2944 (6.4)	106 (3.6)	0.51 (0.34–0.77)		0.45 (0.29–0.7)		0.45 (0.29–0.70)	
	ZAM3 (rural)	2516 (5.5)	59 (2.3)	0.37 (0.24–0.56)		0.32 (0.20–0.51)		0.31 (0.19–0.50)	
	ZAM4 (rural)	1302 (2.8)	26 (2.0)	0.28 (0.16–0.51)		0.26 (0.13–0.51)		0.24 (0.12–0.48)	
	ZAM5	2844 (6.2)	79 (2.8)	0.41 (0.27–0.62)		0.41 (0.26–0.66)		0.38 (0.24–0.61)	
	ZAM6	2961 (6.5)	63 (2.1)	0.34 (0.22–0.53)		0.31 (0.19–0.50)		0.29 (0.18–0.48)	
	ZAM7	3540 (7.7)	94 (2.7)	0.43 (0.28–0.64)		0.46 (0.29–0.72)		0.45 (0.28–0.70)	
	ZAM8	3646 (8.0)	78 (2.1)	0.33 (0.22–0.52)		0.38 (0.24–0.60)		0.36 (0.22–0.59)	
	ZAM9	2145 (4.7)	56 (2.6)	0.39 (0.25–0.61)		0.26 (0.16–0.43)		0.27 (0.16–0.46)	
	ZAM10	2392 (5.2)	59 (2.5)	0.37 (0.24–0.57)		0.48 (0.29–0.78)		0.48 (0.27–0.85)	
	ZAM11	3092 (6.8)	94 (3.0)	0.48 (0.31–0.72)		0.31 (0.20–0.50)		0.31 (0.19–0.49)	
	ZAM12	3401 (7.4)	82 (2.4)	0.39 (0.25–0.58)		0.26 (0.16–0.40)		0.25 (0.16–0.40)	
	ZAM13	3032 (6.6)	73 (2.4)	0.35 (0.23–0.54)		0.28 (0.18–0.44)		0.29 (0.18–0.45)	
	ZAM14	2760 (6.0)	58 (2.1)	0.32 (0.20–0.49)		0.37 (0.23–0.61)		0.35 (0.21–0.57)	
	ZAM15 (rural)	2865 (6.3)	64 (2.2)	0.36 (0.22–0.59)		0.33 (0.19–0.56)		0.34 (0.20–0.59)	
	ZAM16 (rural)	2652 (5.8)	56 (2.1)	0.33 (0.21–0.53)		0.35 (0.21–0.58)		0.38 (0.22–0.63)	

DM = diabetes mellitus; CI = confidence interval; OR = odds ratio; TFT = likelihood ratio test for trend with exposure as a linear variable; All analyses accounted for the two-stage clustered sampling design through use of a logistic regression model with random effects for enumeration area and inclusion of community as a fixed-effect.

^+^ 41,475 participants included in analysis, adjusted for age, sex, household socio-economic position, education, smoking, HIV status and community.

^++^ 38,250 participants included in analysis, adjusted for all variables shown; Missing data: 524 for age, 3883 for HIV status, 3536 for BMI.

# Likelihood ratio tests.

## Tests for linear trend and departure from linearity calculated with underweight as the baseline group.

⁎ Defined as a random blood glucose concentration of ⩾11.1 mmol/L or <11.1 mmol/L with self-reported previous DM diagnosis.

⁎⁎ Based on serology plus self-report for those with no available serology.

⁎⁎⁎ Grouped according to the International BMI Classification.

**Table 2:** Prevalence of diabetes mellitus in the Western Cape sites, with corresponding unadjusted and adjusted odds ratios estimated by logistic regression, showing adjustment for distal risk factors (without measure of adiposity) and more proximal factor (body mass index).

Characteristic		Total number (%)	Number (%) with DM⁎	Unadjusted OR (95% CI)	*P*-value#	Adjusted OR excluding BMI (95% CI)+	*P*-value#	Adjusted OR including BMI (95% CI)++	*P*-value#
Overall		12,496 (100)	1179 (9.4)						

Age (years)	18–24	3207 (25.7)	115 (3.6)	1	<0.001 (TFT *p* < 0.001)	1	<0.001 (TFT *p* < 0.001)	1	<0.001 (TFT *p* < 0.001)
25–29	1810 (14.5)	78 (4.3)	1.21 (0.90–1.63)	1.30 (0.96–1.76)	1.16 (0.86–1.58)
30–34	1456 (11.7)	75 (5.2)	1.48 (1.10–2.00)	1.59 (1.17-2.17)	1.41 (1.03–1.93)
35–39	1290 (10.3)	103 (8.0)	2.34 (1.78–3.09)	2.40 (1.80–3.19)	2.03 (1.51–2.72)
40–49	2090 (16.7)	240 (11.5)	3.48 (2.76–4.39)	3.40 (2.65–4.37)	2.88 (2.22–3.72)
50–59	1454 (11.6)	263 (18.1)	5.94 (4.71–7.50)	5.37 (4.12–6.99)	4.36 (3.32–5.72)
60+	1180 (9.5)	305 (25.9)	9.95 (7.88–12.56)	9.30 (7.07–12.25)	7.63 (5.75–10.12)

Sex	Male	4155 (33.3)	251 (6.0)	1	<0.001	1	<0.001	1	<0.001
Female	8341 (66.7)	928 (11.1)	1.97 (1.70–2.28)	1.95 (1.65–2.30)	1.62 (1.35–1.93)

Household socio-economic position	Very low	2823 (22.6)	188 (6.7)	1	<0.001 (TFT *p* < 0.001)	1	0.149 (TFT *p* = 0.067)	1	0.287 (TFT *p* = 0.153)
Low	3350 (26.8)	298 (8.9)	1.35 (1.08–1.67)	1.26 (1.00–1.58)	1.21 (0.96–1.53)
Medium	3468 (27.8)	362 (10.4)	1.57 (1.26–1.96)	1.26 (0.99–1.60)	1.25 (0.98–1.59)
High	2855 (22.9)	331 (11.6)	1.61 (1.27–2.04)	1.33 (1.03–1.71)	1.25 (0.96–1.61)

Highest level of education	None/grade 1–2	940 (7.5)	151 (16.1)	1	<0.001 (TFT *p* < 0.001)	1	0.341 (TFT *p* = 0.483)	1	0.371 (TFT *p* = 0.231)
Grade 3–6	1876 (15.0)	275 (14.7)	0.85 (0.68–1.06)	1.05 (0.82–1.33)	1.00 (0.78–1.28)
Grade 7–10	4973 (39.8)	503 (10.1)	0.55 (0.45–0.68)	1.05 (0.84–1.33)	0.98 (0.77–1.23)
Grade 11–12	4296 (34.4)	226 (5.3)	0.27 (0.22–0.34)	0.87 (0.66–1.15)	0.81 (0.61–1.08)
College/University	411 (3.3)	24 (5.8)	0.28 (0.18–0.45)	1.10 (0.67–1.80)	1.02 (0.62–1.69)

Smoking history	Never	9248 (74.0)	937 (10.1)	1	<0.001 (TFT *p* < 0.001)	1	0.004 (TFT *p* = 0.002)	1	0.041 (TFT *p* = 0.090)
Ex-smoker	2406 (19.3)	204 (8.5)	0.69 (0.59–0.82)	0.87 (0.72–1.06)	1.01 (0.83–1.23)
Current smoker	842 (6.7)	38 (4.5)	0.44 (0.31–0.62)	0.57 (0.40–0.82)	0.63 (0.43–0.92)

HIV status⁎⁎	Negative	9610 (82.1)	983 (10.2)	1	<0.001	1	<0.001	1	0.008
Positive	2098 (17.9)	124 (5.9)	0.57 (0.47–0.69)	0.69 (0.57–0.85)	0.76 (0.62–0.93)

Body Mass Index⁎⁎⁎ (weight(kg)/height^2^(m))	Recommended weight (18.5–24.9)	4732 (39.0)	251 (5.3)	1	<0.001 (TFT *p* < 0.001)##			1	<0.001 (TFT *p* < 0.001)##
Underweight (<18.5)	678 (5.6)	37 (5.5)	1.00 (0.70–1.43)		0.87 (0.60–1.27)
Overweight (25–29.9)	2859 (23.6)	298 (10.4)	2.09 (1.75–2.49)		1.47 (1.21–1.78)
Obese (⩾30)	3862 (31.8)	549 (14.2)	3.00 (2.56–3.51)		1.78 (1.47–2.14)

Community	WC1	1258 (10.1)	169 (13.4)	1	<0.001	1	<0.001	1	<0.001
WC2	2440 (19.5)	266 (10.9)	0.78 (0.56–1.07)	0.96 (0.68–1.35)	0.96 (0.69–1.36)
WC3 (rural)	212 (1.7)	12 (5.7)	0.36 (0.18–0.74)	0.70 (0.33–1.51)	0.72 (0.34–1.56)
WC4	1809 (14.5)	186 (10.3)	0.76 (0.54–1.05)	1.27 (0.89–1.80)	1.25 (0.88-1.78)
WC5	1275 (10.2)	59 (4.6)	0.32 (0.21–0.48)	0.47 (0.30–0.72)	0.48 (0.31–0.74)
WC6 (rural)	2684 (21.5)	209 (7.8)	0.56 (0.41–0.77)	0.57 (0.41–0.80)	0.56 (0.40–0.79)
WC7	1702 (13.6)	129 (7.6)	0.57 (0.40–0.80)	0.75 (0.52–1.09)	0.73 (0.51–1.07)
WC8	1116 (8.9)	149 (13.4)	1.02 (0.74–1.42)	1.28 (0.90–1.81)	1.30 (0.91–1.85)

DM = diabetes mellitus; CI = confidence interval; OR = odds ratio; TFT = likelihood ratio test for trend with exposure as a linear variable; DFL = likelihood ratio test for departure from linear trend; All analyses accounted for the two-stage clustered sampling design through use of a logistic regression model with random effects for enumeration area and inclusion of community as a fixed effect.

+ 11,699 participants included in analyses, adjusted for age, sex, household socio-economic position, education level, smoking history and HIV status.

++ 11,365 participants included in analysis, adjusted for all variables shown; Missing data: 9 for age, 788 for HIV status, 365 for BMI.

# Likelihood ratio tests.

## Tests for linear trend and departure from linearity calculated with underweight as the baseline group.

⁎ Defined as a random blood glucose concentration of ⩾11.1 mmol/L or <11.1 mmol/L but with self-reported previous DM diagnosis.

⁎⁎ Based on serology plus self-report for those with no available serology.

⁎⁎⁎ Grouped according to the International BMI Classification.

**Table 3:** Prevalence of undiagnosed diabetes among all those identified to have diabetes stratified by participant characteristics.

Characteristic		Zambian sites		Western Cape sites	
Number (%) with undiagnosed DM	*P*-value⁎	Number (%) with undiagnosed DM	*P*-value⁎
Overall		459 (34.5)	–	150 (12.7)	–
Age (years)	18–24	59 (29.2)	0.596	5 (4.4)	0.007
25–29	35 (32.7)	4 (5.1)
30–34	45 (36.9)	6 (8.0)
35–39	36 (34.0)	12 (11.7)
40–49	80 (34.5)	39 (16.3)
50–59	95 (34.3)	38 (14.5)
60+	98 (38.3)	46 (15.1)

Sex	Male	128 (31.8)	0.160	41 (16.3)	0.053
Female	331 (35.8)	109 (11.8)

Household socio-economic position	Very low	100 (43.7)	<0.001	24 (12.8)	0.946
Low	130 (39.3)	35 (11.7)
Medium	100 (27.3)	48 (13.3)
High	129 (32.0)	43 (13.0

Highest level of education	None/grade 1–2	69 (48.6)	<0.001	28 (18.5)	0.147
Grade 3–6	79 (41.4)	32 (11.6)
Grade 7–10	211 (32.9)	63 (12.5)
Grade 11–12	59 (29.4)	26 (11.5)
College/University	41 (26.8)	1 (4.2)

Smoking history	Never	391 (33.4)	0.042	125 (13.3)	0.249
Ex-smoker	24 (34.3)	19 (9.3)
Current smoker	44 (49.4)	6 (15.8)

Body Mass Index⁎⁎⁎	Recommended weight (18.5–24.9)	166 (30.9)	0.298	19 (7.6)	0.047
Underweight (<18.5)	38 (38.8)	5 (13.5)
Overweight (25–29.9)	115 (35.2)	44 (14.8)
Obese (⩾30)	95 (35.5)	77 (14.0)

HIV status⁎⁎	Negative	329 (34.7)	0.100	131 (13.3)	0.098
Positive	58 (28.7)	10 (8.1)

Self-reported current tuberculosis	No	455 (34.5)	0.898	147 (12.6)	0.146
Yes	4 (36.4)	3 (27.7)

Community	ZAM1	33 (11.7)	<0.001	–	–
ZAM2	27 (25.5)	–
ZAM3 (rural)	21 (35.6)	–
ZAM4 (rural)	15 (57.7)	–
ZAM5	25 (31.7)	–
ZAM6	27 (42.9)	–
ZAM7	46 (48.9)	–
ZAM8	43 (55.1)	–
ZAM9	25 (44.6)	–
ZAM10	15 (25.4)	–
ZAM11	35 (37.2)	–
ZAM12	32 (39.0)	–
ZAM13	28 (38.4)	–
ZAM14	33 (56.9)	–
ZAM15 (rural)	38 (59.4)	–
ZAM16 (rural)	16 (28.6)	–
WC1	–	–	19 (11.2)	0.007
WC2	–	33 (12.4)
WC3 (rural)	–	1 (8.3)
WC4	–	13 (7.0)
WC5	–	15 (25.4)
WC6 (rural)	–	36 (17.2)
WC7	–	18 (14.0)
WC8	–	15 (12.7)

DM = diabetes mellitus.

⁎ Pearson’s Chi-squared tests; Total number of participants identified to have diabetes and therefore included in these analyses = 1329 in Zambia and 1179 in Western Cape.

⁎⁎ Based on serology plus self-report for those with no available serology.

⁎⁎⁎ Grouped according to the International BMI Classification.

**Table 4:** Proportion of those with known diabetes on diabetes treatment.

Diabetes treatment		Zambian sites	Western Cape sites
Number (%) of those with known diabetes	
Any treatment		296 (34.0)	418 (40.6)
Dietary	38 (4.4)	5 (0.5)
Hypoglycaemic tablets	210 (24.1)	338 (32.9)
Insulin	48 (5.5)	75 (7.3)
None		574 (66.0)	611 (59.4)

**Table 5:** Random blood glucose concentration among those with known diabetes.

Random blood glucose concentration (mmol/L)	Number (%) of those with known diabetes		
All participants	Participants on diabetes treatment	Participants not on diabetes treatment
Zambian sites			
<6.0	385 (44.3)	48 (16.2)	337 (58.7)
6.0–7.8	186 (21.4)	38 (12.8)	148 (25.8)
⩾7.8	299 (34.4)	210 (71.0)	89 (15.5)

Western Cape sites			
<6.0	456 (44.3)	89 (21.3)	367 (60.1)
6.0–7.8	237 (23.0)	73 (17.5)	164 (26.8)
⩾7.8	336 (32.7)	256 (61.2)	80 (13.1)

Categorisation of glucose concentration based on International Diabetes Federation guidelines for target pre-prandial glucose concentration (<6.0 mmol/L) and target postprandial glucose concentration (<7.8 mmol/L) [18].
