# Supplementary material for: Diabetes mellitus in Zambia and the Western Cape province of South Africa: Prevalence, risk factors, diagnosis and management
Source: Diabetes Res Clin Pract. 2016 Aug;118:1–11. doi: 10.1016/j.diabres.2016.05.001 (PMC4994576; doi:10.1016/j.diabres.2016.05.001)
Supplement: Supplementary data 1 [file mmc1.docx]

Supplementary table: Baseline characteristics of individuals excluded due to missing data for glycaemia

|  | | Number (%) | |
| --- | --- | --- | --- |
| Characteristic | | **Zambia** | **Western Cape** |
| Total |  | 12,042 (100) | 20,296 (100) |
| Age (years) | 18-24 | 4,438 (37.5) | 6,322 (31.2) |
|  | 25-29 | 2,082 (17.6) | 3,875 (19.1) |
|  | 30-34 | 1,544 (13.1) | 2,830 (14.0) |
|  | 35-39 | 1,078 (9.1) | 2,170 (10.7) |
|  | 40-49 | 1,248 (10.6) | 2,686 (13.2) |
|  | 50-59 | 717 (6.1) | 1,472 (7.3) |
|  | 60+ | 725 (6.1) | 928 (4.6) |
| Sex | Male | 4,334 (36.0) | 8,260 (40.7) |
|  | Female | 7,708 (64.0) | 12,036 (59.3) |
| Household socio-economic position | Very low | 3,367 (28.0) | 5,747 (28.3) |
|  | Low | 3,186 (26.5) | 4,707 (23.2) |
|  | Medium | 2,858 (23.7) | 4,938 (24.3) |
|  | High | 2,631 (21.9) | 4,904 (24.2) |
| Highest level of education | None/ grade 1-2 | 1,032 (8.6) | 899 (4.4) |
|  | Grade 3-6 | 1,597 (13.3) | 2,009 (9.9) |
|  | Grade 7-10 | 5,899 (49.0) | 7,331 (36.1) |
|  | Grade 11-12 | 2,682 (22.3) | 9,089 (44.8) |
|  | College/ University | 832 (6.9) | 968 (4.8) |
| Smoking history | Never | 10,462 (86.9) | 15,454 (76.1) |
|  | Ex-smoker | 502 (4.2) | 2,534 (12.5) |
|  | Current smoker | 1,078 (9.0) | 2,308 (11.4) |
| HIV status^#^ | Negative | 5,078 (84.7) | 7,250 (85.9) |
|  | Positive | 917 (15.3) | 1,193 (14.1) |
| BMI* | Recommended weight (18.5-24.9) | 6,446 (67.6) | 119 (49.8) |
|  | Underweight (<18.5) | 1,091 (11.4) | 16 (6.7) |
|  | Overweight (25-29.9) | 1,472 (15.4) | 51 (21.3) |
|  | Obese (≥30) | 525 (5.5) | 53 (22.1) |
| Community | ZAM1 | 305 (2.5) | - |
|  | ZAM2 | 960 (8.0) | - |
|  | ZAM3 (rural) | 1,483 (12.3) | - |
|  | ZAM4 (rural) | 364 (3.0) | - |
|  | ZAM5 | 965 (8.0) | - |
|  | ZAM6 | 541 (4.5) | - |
|  | ZAM7 | 420 (3.5) | - |
|  | ZAM8 | 293 (2.4) | - |
|  | ZAM9 | 1,067 (8.9) | - |
|  | ZAM10 | 1,551 (12.9) | - |
|  | ZAM11 | 785 (6.5) | - |
|  | ZAM12 | 431 (3.6) | - |
|  | ZAM13 | 837 (7.0) | - |
|  | ZAM14 | 1,166 (9.7) | - |
|  | ZAM15 (rural) | 257 (2.1) | - |
|  | ZAM16 (rural) | 617 (5.1) | - |
|  | WC1 | - | 2,817 (13.9) |
|  | WC2 | - | 1,497 (7.4) |
|  | WC3 (rural) | - | 3,933 (19.4) |
|  | WC4 | - | 2,365 (11.7) |
|  | WC5 | - | 2,801 (13.8) |
|  | WC6 (rural) | - | 1,392 (6.9) |
|  | WC7 | - | 2,437 (12.0) |
|  | WC8 | - | 3,054 (15.1) |

*BMI = body mass index ((weight(kg)/height^2^(m));  ^#^Based on serology plus self-report for those with no available serology; *Grouped according to the International BMI Classification.*
